# The Rise of Plant Pan‐Genomes: From Genome Variation to Predictive Breeding

**DOI:** 10.1002/ggn2.70044

**Published:** 2026-09-01

**Authors:** Dan Peng, Xuezhu Liao, Liang Tang, Zhiqiang Wu

**Affiliations:** ^1^ State Key Laboratory of Tropical Crop Breeding Shenzhen Branch, Guangdong Laboratory of Lingnan Modern Agriculture Key Laboratory of Synthetic Biology Ministry of Agriculture and Rural Affairs Agricultural Genomics Institute at Shenzhen Chinese Academy of Agricultural Sciences Shenzhen China; ^2^ School of Ecology Hainan University Haikou China; ^3^ International Joint Center For Terrestrial Biodiversity Around South China Sea of Hainan Province School of Ecology Hainan University Haikou China

## Abstract

Plant pan‐genomics is entering a new phase beyond genome variation discovery, requiring a shift from cataloguing genomic diversity toward understanding how variation generates biological function and breeding value. Here, we propose that the future of plant pan‐genomics will be shaped by three conceptual transitions. First, structural variation (SV), presence–absence variation (PAV), and haplotype diversity should be interpreted not merely as genomic differences, but as regulatory components that influence gene networks, chromatin organization, and complex traits. Second, the expansion from species‐level pan‐genomes to genus‐level super pan‐genomes provides an evolutionary framework for uncovering adaptive genetic modules preserved in wild relatives and overlooked during domestication. Third, integrating pan‐genomes with pan‐omics, three‐dimensional genome analyses, and artificial intelligence will enable the transformation of genomic variation into predictive models for crop improvement. We further propose that the ultimate value of pan‐genomes lies not in generating increasingly complete genome collections, but in establishing a mechanistic bridge between genome diversity, biological function, and breeding decisions. This transition will move crop improvement from empirical selection toward rational genome design, where evolutionary diversity can be systematically interpreted, predicted, and engineered.

## Graphical abstract

Plant pan‐genomics is shifting from genome variation discovery to understanding how diversity drives biological function and breeding value. Future advances will integrate SV/PAV, haplotype diversity, super pan‐genomes, pan‐omics, 3D genomics, and AI to transform genomic resources into predictive breeding strategies. The goal is to establish a mechanistic link between genome diversity, function, and crop improvement, enabling genome design and selection.

## 1 Introduction

Over the past decade, plant genomes have been radically transformed by advances in long‐read sequencing, chromosome‐scale assembly, and population‐scale genome analysis. Prior to this shift, research had been centered on a single reference genome, an approach that represented complex species diversity within a single linear framework. However, as genome research has progressed and population‐scale sequencing has become increasingly accessible, the limitations of this model have become increasingly apparent. A single linear reference cannot adequately accommodate widespread gene presence–absence variation (PAV), structural rearrangements, and differences in gene dosage within species, nor can it fully reflect the breadth of genetic potential underlying phenotypic diversity.

The emergence of pan‐genome provides researchers with a more comprehensive tool to explore how genetic variations influence plant genetic diversity, genome evolution, and the mechanisms regulating complex traits. By systematically constructing species‐level genome collections, pan‐genome reveals a dynamic genetic landscape composed of both core and variable genes. In several crop species, variable genes account for a substantial proportion of the total gene repertoire, indicating that the genetic identity of a species extends far beyond what any single genome can define [1]. Based on a systematic review of nearly one hundred highly cited and representative studies published between 2020 and 2026, we summarize the developmental trajectory of plant pan‐genome, highlight the key breakthroughs it has enabled in understanding genomic diversity and functional variation, and discuss its far‐reaching implications for future crop improvement (Supporting Information Table S1).

## 2 Progress From a Single Reference to Complete Genomes

**FIGURE 1:**
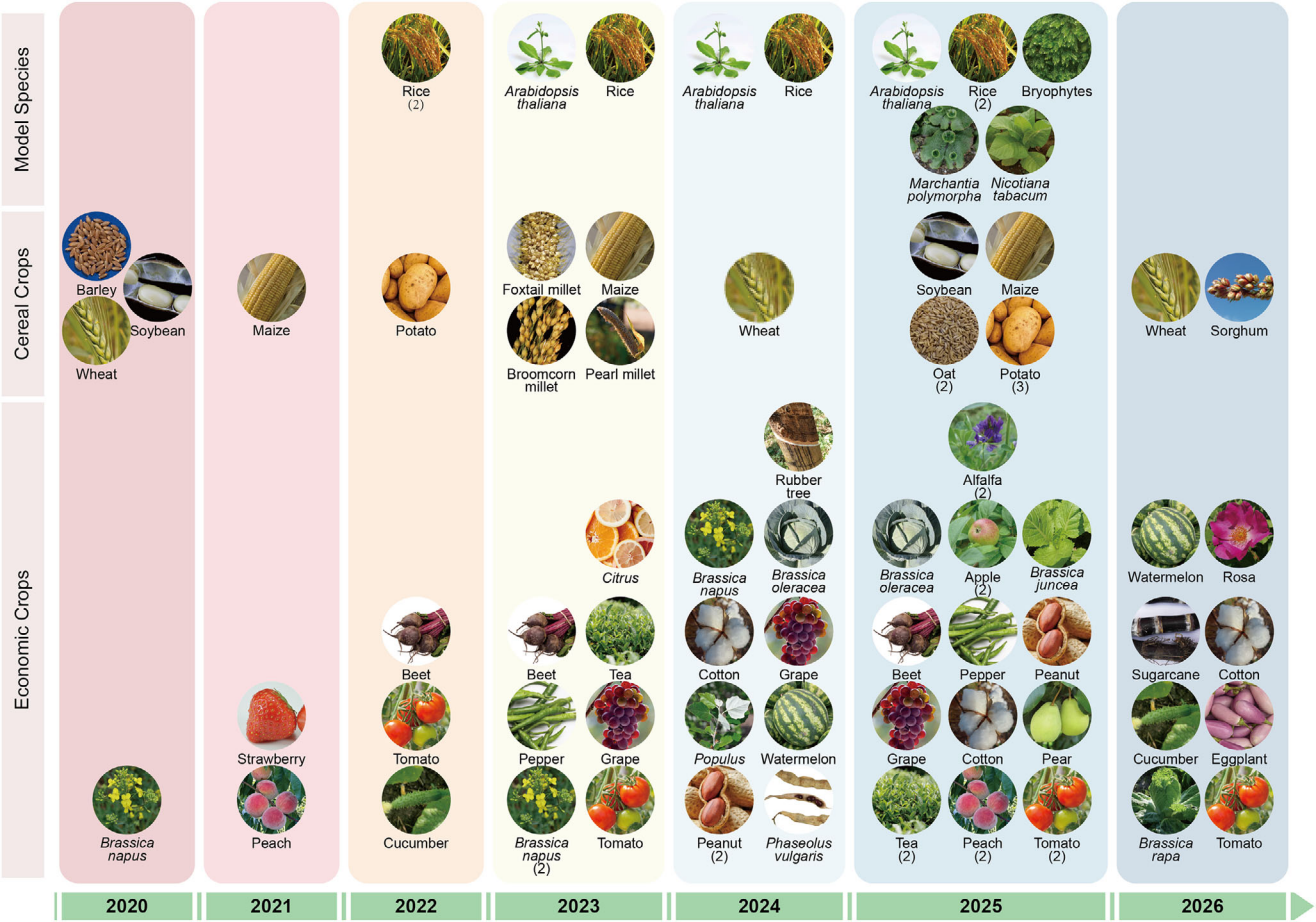
Developmental trajectory and taxonomic expansion of plant pan‐genome studies from 2020 to 2026. Timeline illustrating the rapid expansion and diversification of plant pan‐genome research from 2020 to 2026. Early studies were primarily focused on model species and major cereal crops, whereas recent efforts increasingly encompass horticultural, industrial, and other economically important species. The figure also reflects the accelerated accumulation of high‐quality pan‐genome resources following advances in long‐read sequencing and genome assembly technologies. Numbers in parentheses indicate multiple representative studies reported for the corresponding species within a given year. Author credits, source URLs, and license information for all reused images are provided in Table S1.

The greatest breakthrough of plant pan‐genomics lies in its ability to greatly expand our understanding of genome diversity. Traditional reference genomes are primarily constructed using a single representative accession, whereas pan‐genomes integrate genomic information from multiple individuals or even closely related species, providing a more comprehensive representation of a species’ genetic information [2, 3, 4, 5]. A major breakthrough emerged after 2020 with the widespread adoption of graph‐based pan‐genomes. Landmark studies in soybean and tomato demonstrated that graph‐based pan‐genomes enable the systematic integration of large‐scale SVs across diverse individuals [6, 7]. Early pan‐genome studies during this stage were primarily focused on model plants and major cereal crops, whereas recent studies have increasingly expanded into horticultural, industrial, and other economically important species [8, 9, 10, 11, 12, 13, 14, 15] (Figure 1). Currently, pan‐genome research is advancing towards a new phase centered on complete genomes. Regions such as centromeres, telomeres, and highly repetitive sequences, which were previously excluded due to technical limitations, are now being resolved through telomere‐to‐telomere (T2T) assemblies across multiple species [16, 17, 18, 19, 20].

The rapid development of this field has largely been driven by continuous advancements in sequencing and assembly technologies [21]. Graph‐based pangenomes are primarily constructed through two complementary strategies: reference‐guided graph expansion and integration of multiple whole‐genome assemblies. Several computational frameworks, including Pangenome Graph Builder (PGGB), Minigraph‐Cactus, and the vg toolkit, have been widely adopted for the construction and analysis of complex genome graphs [22, 23, 24]. These approaches have enabled researchers to overcome several fundamental limitations associated with linear reference genomes. First, early population genomics studies largely relied on large‐scale short‐read resequencing, in which sequencing reads were aligned to a single reference genome to identify SNPs, small insertions/deletions, and a limited number of PAVs. Although these approaches have greatly expanded genetic information associated with reference genomes and remain widely used, they are inherently limited in resolving genomic sequences absent from the reference. Due to the short length of resequencing reads, non‐reference sequences cannot be accurately characterized in terms of their sequence length, internal composition, or the presence of genes and regulatory elements [25, 26, 27]. Consequently, the structural relationships among such sequences across different individuals remain difficult to determine. By integrating alternative sequences from multiple individuals, pangenomes preserve rare genomic fragments present only in a subset of accessions, often referred to as “ghost sequences”, allowing previously undetectable, unlocalized, or functionally unannotated genetic variants to be incorporated into population genetic analyses and trait association studies [28].

Second, highly repetitive regions, large‐scale structural variants, and highly divergent genomic segments often lead to alignment failures and inaccurate breakpoint resolution, making them difficult to characterize within a single reference framework. By integrating multiple high‐quality genome assemblies, pangenomes provide a unified framework that incorporates alternative sequences and structural variants across individuals, enabling more comprehensive representation of highly variable genomic regions, including gene cluster expansions, complex rearrangements, and non‐reference genes [29, 30]. These advances substantially expand the spectrum of genetic variation available for population genomic analyses and genomic prediction, providing a foundation for dissecting the functional consequences of structural variation and its contribution to complex trait variation.

## 3 Expansion From Species to Genus‐Level Super Pan‐Genomes

**FIGURE 2:**
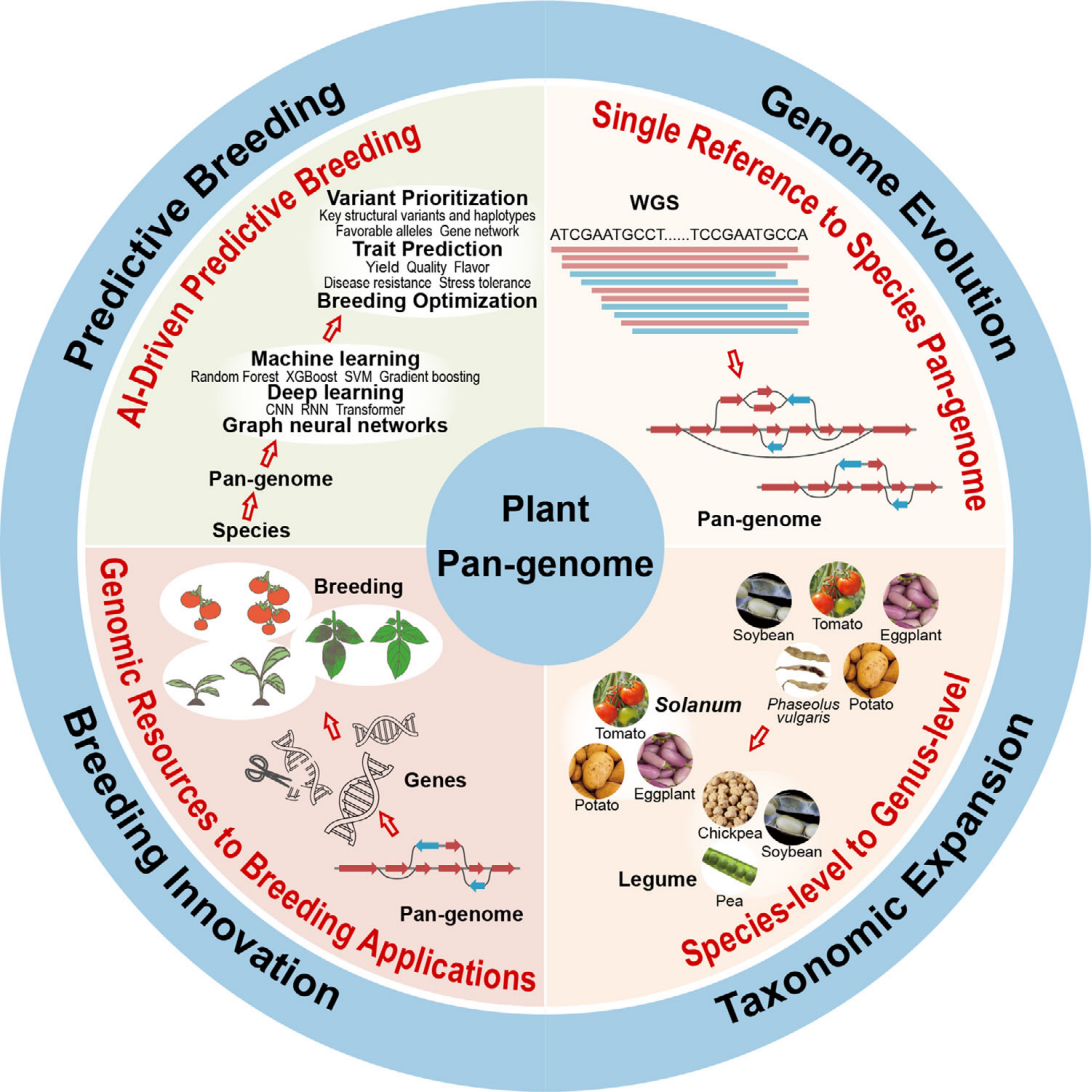
Schematic overview of the major developmental directions and applications of plant pan‐genome research. The outer ring summarizes the broader conceptual advances enabled by pan‐genome research, including genome evolution, taxonomic expansion, breeding innovation, and predictive breeding, whereas the inner red labels represent the major developmental directions and technological transitions within each domain. Pan‐genomes have evolved from single‐reference genome frameworks toward graph‐based and species‐level pan‐genome representations, enabling comprehensive characterization of structural variations and genome evolution. Expansion from species‐level analyses to genus‐level super pan‐genomes facilitates the exploration of adaptive diversity and evolutionary relationships across related taxa. Simultaneously, pan‐genome resources are increasingly being translated into breeding applications through the identification of functional genes and favorable alleles. Integration with artificial intelligence and machine learning approaches further supports variant prioritization, trait prediction, and breeding optimization, ultimately promoting the transition toward AI‐driven predictive breeding and genome design.

Another notable trend in plant pan‐genome has been the expansion of analytical scope from individual species to the genus level. Species‐level pangenomes primarily characterize the composition and distribution of intraspecific genetic diversity, whereas genus‐level super‐pangenomes extend this framework into a phylogenetic context to investigate the evolutionary processes underlying the emergence, retention, and loss of genetic variation. By integrating genomic resources from multiple closely related species, super‐pangenomes enable the tracing of ancestral genetic components across divergent lineages, revealing the evolutionary trajectories of genome diversification associated with speciation, adaptation, and domestication (Figure 2).

Adaptive genetic diversity is not uniformly retained throughout evolution [28]. In particular, modern cultivated species have lost substantial genetic variation through domestication, artificial selection, and bottleneck effects, whereas many important alleles associated with environmental adaptation are still preserved in wild relatives [8, 31, 32, 33]. Genus‐level super pan‐genomes have revealed that overcoming the genetic bottleneck in modern crops may depend on long‐neglected genetic diversity from wild relatives [34]. These wild species harbor abundant adaptive genetic modules that have remained largely untouched by domestication. These wild and related species represent genomic states accumulated over long evolutionary trajectories across distinct lineages. By comparing lineage‐specific patterns of gene gain and loss, structural variation accumulation, and the divergence of adaptation‐associated genomic regions, super‐pangenomes can identify ancestral genetic components that have been lost during crop domestication and reveal the evolutionary history of these genetic modules under the combined influences of natural and artificial selection. For example, a recent rose super‐pangenome study uncovered extensive introgression events among different taxonomic groups, demonstrating long‐term genetic exchange and hybridization within the genus [15]. These cross‐lineage genetic exchanges have not only shaped the genomic composition of modern roses but have also provided new insights into speciation, lineage diversification, and the complex reticulate evolutionary history of roses. Similarly, an oat super‐pangenome study revealed that a large proportion of newly identified haplotypes originated from wild lineages, indicating that wild relatives are not simply genetic backgrounds eliminated during domestication, but instead preserve genetic diversity shaped by distinct evolutionary histories [35]. Together, these findings highlight that domestication is not a linear process but is instead accompanied by gene flow, extensive hybridization, and genomic recombination [36, 37]. Genus‐level super‐pangenomes provide a framework for understanding how genetic variation emerges, evolves, and is maintained across evolutionary lineages, while enabling the re‐utilization of evolutionary genetic resources accumulated over long timescales.

Beyond integrating adaptive allelic variation from related species, super pan‐genomes establish comparative frameworks at population and cross‐species scales, enabling previously inaccessible highly repetitive genomic regions to be investigated through comparative genomics. Centromeres represent one of the most prominent examples of such regions. Numerous studies have demonstrated that centromeres can evolve rapidly, even among closely related species or varieties [38, 39]. Unlike traditional studies that focus on individual reference centromeres, pan‐centromere analyses integrate complete centromere sequences from multiple individuals and closely related species, providing a systematic framework to characterize centromere diversity, evolution, and conservation at both population and species levels. Recent studies in Brassicaceae, tomato, and rice have adopted pangenome‐based approaches to resolve complex centromere architectures and reconstruct centromere evolution among populations and related species [17, 40, 41]. Comparative analyses have revealed substantial differences in centromere size, satellite repeat composition, retrotransposon landscapes, and centromere repositioning among species and populations, indicating that although centromeres maintain conserved functions in chromosome segregation, their structures undergo rapid and dynamic evolution. However, current pan‐centromere studies remain relatively limited in scope. Most advances in pan‐centromere research have been enabled by the development of T2T genome assemblies, and current analyses largely focus on sequence‐level variation, including centromeric element composition, copy number variation, and LTR retrotransposon abundance. Thus, these studies largely remain within the framework of conventional comparative genomics and have not yet fully explored how pangenome‐level structural variations, including SVs and PAVs, contribute to centromere diversity and evolutionary dynamics. Deciphering the connections between genome structural variation and centromere evolution represents an important future direction for pan‐centromere research.

## 4 From Genetic Diversity to Breeding Innovation

The primary goal of pan‐genomics, both historically and currently, has been to construct more complete and representative genomic resources and to uncover additional genetic variation. Currently, plant pan‐genome research is undergoing a critical transition, where our ability to identify large‐scale structural variations (SVs) has far exceeded our ability to interpret their biological functions and breeding values. The major challenge is to determine which variants are functionally relevant, how they contribute to phenotypic diversity, and how these insights can be integrated into predictive breeding frameworks.

Traditional linear reference genomes can identify differences between a given accession and the reference sequence; however, in studies involving closely related species or genus‐level comparisons, highly divergent genomic regions are often overlooked due to substantial sequence divergence [42]. By integrating multiple high‐quality genomes, pan‐genomes organize alternative sequences, missing regions, and complex structural variations from different accessions into a unified framework, enabling researchers to move beyond “individual variant detection” toward the characterization of comprehensive “structural variation landscapes.” For example, in cabbage, a graph‐based pan‐genome constructed from 704 accessions and 27 representative genomes systematically characterized population‐scale SV diversity and revealed that some SVs were not merely sequence differences, but instead contributed to morphological variation by altering DNA methylation levels, transcription factor binding sites, and gene expression patterns [43]. This study demonstrates that the contribution of pan‐genomics does not simply lie in the discovery of SVs themselves, but rather in placing SVs within population genetic contexts and regulatory networks to reveal how structural variations contribute to complex trait formation.

This advantage is particularly important for understanding how structural variations contribute to phenotypic diversity. Pan‐genomes are increasingly being transformed into functional breeding platforms that connect genomic variation with trait prediction, breeding optimization, and ultimately predictive crop improvement (Figure 2). The ultimate contribution of pan‐genome lies in its ability to provide evidence‐based and actionable guidance for breeding programmes that would otherwise rely heavily on empirical screening.

Model‐based prediction represents one of the emerging approaches for translating pan‐genome information into practical applications. Traditional genomic selection (GS) primarily relies on SNP markers to construct prediction models. However, SNPs generally capture genetic signals indirectly through linkage with causal variants and are insufficient to fully represent the effects of gene presence–absence variations, gene dosage changes, and large‐scale regulatory region rearrangements. Furthermore, the accumulation of deleterious mutations and the so‐called “broken window effect” highlight that genome architecture itself represents an important functional dimension [44]. Therefore, incorporating pan‐genome‐derived variation into genomic prediction models has the potential to improve the prediction accuracy of complex traits, particularly for traits such as disease resistance, environmental adaptation, and yield that are strongly influenced by structural variations. Empirically, incorporating SVs into genomic selection models has significantly improved prediction accuracy in grape [30]. These advances signal that breeding is approaching the concept of “digital twin breeding”, in which the phenotypic outcomes of thousands of genetic combinations can be simulated computationally before experimental validation.

Even more forward‐looking is the concept of modular disease‐resistance breeding. A recent concept in potato demonstrated that an immune receptor domain from one species can be ‘plugged’ into another NLR to broaden its recognition spectrum [45]. This suggests that, based on genus‐level pan‐genome element libraries, immune receptor domains derived from diverse species may be rationally combined to address emerging pests and pathogens under global climate change. Such strategies resemble integrated circuit design, where functional modules are assembled to generate novel traits that may not naturally occur in existing germplasm.

## 5 Looking Ahead to Crop Science in the Pan‐Genome Era

Research on linear genomes is no longer limited to constructing more complete genome resources but is increasingly expanding toward the three‐dimensional genome dimension [46, 47]. Integrating pan‐genome variation with three‐dimensional chromatin architecture provides an opportunity to reveal how structural variations are transmitted from the DNA level to regulatory processes and how they contribute to complex traits and environmental adaptation. Increasing evidence indicates that structural variations are not merely differences at the genomic sequence level, but can also influence gene regulation by reshaping three‐dimensional chromatin folding patterns, long‐range enhancer–promoter interactions, and local chromatin environments. The pan‐genome and pan‐three‐dimensional genome studies in cotton provide direct evidence for this concept [48]. By integrating genome assemblies, Hi‐C, and transcriptome data from ten diploid cotton species, these studies linked structural variation, chromatin architectural changes, and differences in fiber quality traits.

Meanwhile, a soybean pan‐3D genome study integrated chromosome‐scale genome assemblies and Hi‐C datasets from multiple accessions to construct a genotype‐resolved three‐dimensional chromatin landscape using the ZH13 reference genome as a unified coordinate framework [49]. The study revealed extensive variation in chromatin compartments among different accessions, with a subset of genomic regions undergoing A/B compartment switching. In addition, TAD boundaries exhibited both conserved and accession‐specific patterns, indicating that genetic variation among accessions not only affects DNA sequences but also reshapes higher‐order chromatin organization. The oat pan‐transcriptome study integrated transcriptomic datasets from multiple genetic backgrounds to characterize expression‐level variation among accessions [50]. By quantifying transcripts against the corresponding reference genomes, this approach established a population‐scale atlas of expression diversity. Together, these studies further extend the concept of pangenomics toward pan‐omics, enabling the integration of genomic variation with multiple molecular layers to better understand how genetic diversity influences regulatory states and phenotypic variation.

With the establishment of increasingly large‐scale population‐level multi‐omics resources, future pan‐omics research will move beyond identifying genetic variation alone and focus on understanding the relationships among different layers of biological information. Pan‐epigenomics and pan‐regulomics further expand our understanding of non‐coding regulatory variation. Epigenetic features, including DNA methylation, histone modifications, and chromatin accessibility, exhibit strong tissue‐ and environment‐specific patterns, while variation in regulatory regions among different genetic backgrounds may explain phenotypic differences that are difficult to capture through conventional genetic analyses. Future efforts should integrate structural variations identified from pangenomes, spatial regulatory information from pan‐3D genomes, and functional states revealed by pan‐transcriptomes and pan‐epigenomes, together with pan‐proteomic, pan‐metabolomic, and functional genomic datasets. Such multi‐layered integration will establish a comprehensive framework linking the emergence of genetic variation, remodeling of regulatory networks, and eventual phenotypic outcomes, providing a new theoretical foundation for complex trait prediction, adaptive evolution studies, and precision breeding.

Translating these genomic resources into actionable breeding decisions will be a major direction for future applications. In practical breeding programs, pangenome information can facilitate prediction at multiple levels. For complex quantitative traits, including yield, plant architecture, flowering time, and quality‐related traits, pangenome‐derived variants can complement traditional SNP markers by capturing genetic effects associated with gene dosage variation, structural variation, and differences in regulatory elements [51, 52, 53, 54]. For traits such as disease resistance, stress tolerance, and environmental adaptation, pangenomes can identify functional haplotypes retained in wild relatives and landraces, providing valuable resources for the discovery of favorable alleles and informed parental selection [55, 56]. Furthermore, integrating pangenome information into genomic prediction models can shift breeding strategies from genetic distance‐based evaluation toward function‐oriented parental selection. In heterosis prediction, pangenomes can reveal complementary relationships between parental genomes based on structural variation and combinations of favorable alleles, rather than relying solely on overall SNP divergence [44]. In mating design, the integration of trait‐associated genetic modules, environmental adaptation information, and predictive models can optimize parental combinations and increase the probability of generating progeny with desired trait combinations, ultimately transforming breeding from empirical crossing toward rational, design‐based breeding.

Finally, the integration of artificial intelligence (AI) with pan‐genomic and pan‐omics resources will become a critical driver for translating genomic knowledge into breeding decisions. Machine learning and deep learning approaches, particularly graph neural network models, can integrate diverse types of genetic variation within pan‐genome frameworks and capture potential nonlinear relationships among genetic variants, regulatory elements, gene expression patterns, environmental responses, and complex phenotypes [57]. By further incorporating pan‐three‐dimensional genomes, pan‐transcriptomes, pan‐epigenomes, and functional genomics data, artificial intelligence models have the potential to learn the mechanisms through which genetic variation shapes phenotypic traits, enabling a transition from “association analysis” toward “functional prediction”.

In addition, the development of generative models and foundation models for genome sequences is further expanding the application boundaries of pan‐genomes. Genome large language models, represented by biological sequence foundation models such as Evo2, further treat DNA sequences as a form of “language” and infer sequence–function relationships from large‐scale pan‐genomic variation datasets, thereby providing a new computational paradigm for gene function prediction and variant prioritization [58]. By integrating multi‐omics datasets and gene regulatory networks, these computational frameworks can improve the prediction of disease resistance, stress tolerance, and environmental adaptation. Such advances are accelerating the translation of pan‐genome resources into breeding innovation by supporting genomic selection, allele optimization, and AI‐assisted predictive breeding strategies. Together, these developments are driving the transition from genome discovery toward genome design, ultimately enabling more precise, scalable, and data‐driven crop improvement (Figure 2).

## 6 Challenges Ahead for Plant Pan ‐ Genome Research

The advancement of pangenomics is driven not only by improvements in genome completeness but also by efforts to increase the representativeness of genomic resources. However, selecting a set of representative genomes that adequately captures the full extent of genetic diversity within a species remains a major challenge.

A similar challenge also exists in linear genome analyses. Researchers often assume that “more individuals” lead to “a better pan‐genome”, but this assumption is not always valid. With the increasing availability of high‐quality genome assemblies, highly related or genetically redundant individuals often provide limited additional information while substantially increasing sequencing costs, graph complexity, and data storage requirements. Meanwhile, excessive inclusion of redundant genomes can dramatically increase computational burdens during downstream pangenome construction and analysis [59, 60]. Therefore, the selection of representative genomes should prioritize maximizing biological diversity rather than maximizing sample numbers.

Effective sampling strategies should consider multiple dimensions of diversity. At the species level, populations formed under different environmental conditions often harbor genetic variants associated with local adaptation. Incorporating accessions with extreme or contrasting phenotypes, which may contain key variants underlying important traits, as well as materials from diverse geographic origins and phenotypic backgrounds, can improve the discovery of adaptive alleles associated with environmental responses [61, 62, 63]. At higher taxonomic levels, phylogenetic diversity becomes a critical consideration because it ensures that sampled genomes cover major evolutionary lineages and reduces information redundancy caused by excessive sampling of closely related individuals [64, 65]. Domestication history represents another important dimension. Modern cultivated varieties have typically lost substantial genetic diversity due to domestication bottlenecks, whereas wild relatives retain important genetic variations that may contribute to stress tolerance and environmental adaptation [32, 66, 67]. Therefore, representative genome selection should aim to maximize biological diversity rather than simply increasing the number of sampled genomes.

Despite rapid advances, plant pan‐genome research still faces several important technical limitations. First, although graph‐based pan‐genomes provide powerful representations of genomic diversity, standardized frameworks for variant detection and genotyping remain lacking, particularly in polyploid and highly heterozygous species [68]. Unlike linear references, graph pan‐genomes must resolve complex alternative paths, multi‐allelic structures, and repetitive regions, and convert diverse structural variants into reliable genotypes suitable for population analyses and genomic prediction [22, 23]. Cross‐species comparisons among closely related species with different chromosome numbers and ploidy levels remain highly challenging, and current computational tools often struggle to accurately process such complex evolutionary relationships. Moreover, most current analyses, including studies of centromeres, epigenetic modifications, and differential gene expression, still rely on mapping WGBS, ATAC‐seq, and RNA‐seq datasets to their respective linear reference genomes, followed by integration with structural variation information [50, 69]. Therefore, these approaches have not yet achieved truly pangenome graph‐based analyses in which the graph pangenome itself serves as the reference framework. In addition, differences in graph construction strategies, variant representation schemes, and genotyping pipelines further limit the integration of cross‐species genomic datasets [70]. The development of more accurate and efficient alignment algorithms, together with standardized frameworks for SV/PAV detection and pangenome variant representation, will be essential for transforming pangenomes from static genomic resources into practical platforms for breeding applications.

Second, integrating multi‐omics data into pan‐genome frameworks remains a major challenge for functional interpretation. Most transcriptomic, epigenomic, and chromatin interaction analyses still rely on linear reference genomes, whereas pan‐genomes represent diversity through graph structures or multiple genome paths [71, 72]. Consequently, accurately assigning RNA‐seq reads, DNA methylation signals, chromatin accessibility profiles, and Hi‐C interactions across alternative genomic structures remains difficult. Developing approaches that enable multi‐omics integration within complex pan‐genome architectures will be critical for linking genomic variation with biological function.

Third, the predictive capability of genome‐based models remains limited. Although incorporating SNPs, SVs, and PAVs into prediction models has improved trait prediction, genomic datasets often contain strong dependencies, population structures, and shared sequences that complicate model evaluation [73]. In particular, distinguishing biologically meaningful associations from spurious correlations and avoiding overfitting remain major challenges [74]. Improving model robustness will require larger and more diverse training populations, independent validation datasets, and better integration of functional genomic information.

## 7 Conclusion

Overall, pan‐genome research demonstrates that species identity is defined not by a single genome but by a dynamic system composed of both core and variable sequences. It reveals that genome evolution is shaped not only by point mutations but also by large‐scale structural rearrangements. More importantly, the pan‐genome provides a new conceptual framework for crop science: moving from reading individual reference genomes toward systematically understanding genetic diversity, and ultimately toward rational genome design. With the continued integration of high‐quality genome assemblies, graph‐based pan‐genomes, evolutionary genomics, and AI‐driven analytical frameworks, pan‐genomes are evolving from static databases into strategic platforms capable of unlocking the full genetic potential of crops. These advances mark the beginning of a new era in agriculture characterized by rational design, precise intervention, and increasingly predictable breeding outcomes.

## Funding

This work was funded by the Chinese Academy of Agricultural Sciences Elite Youth Program (110243160001007).

## Conflicts of Interest

The authors declare no conflicts of interest.

## Supporting information




**Supporting File 1**: ggn270044‐sup‐0001‐SuppMat.xlsx.

**Supporting File 2**: ggn270044‐sup‐0002‐Table1.xlsx.

## Data Availability

The data that supports the findings of this study are available in the supplementary material of this article.
